# Alginate encapsulated cell blocks improve diagnostic consistency and long term specimen integrity in routine pathological diagnostics and cancer research

**DOI:** 10.1038/s41598-025-21615-0

**Published:** 2025-10-28

**Authors:** Jędrzej Borowczak, Monika Kula, Karol Gostomczyk, Katarzyna Olejnik, Magdalena Drozd, Magdalena Kostrzewska-Poczekaj, Małgorzata Jarmuż-Szymczak, Małgorzata Wierzbicka, Maciej Giefing, Łukasz Szylberg, Andrzej Marszałek, Magdalena Bodnar

**Affiliations:** 1 Clinical Department of Oncology, Oncology Centre – Prof. Franciszek Łukaszczyk Memorial Hospital, 85-796 Bydgoszcz, Poland; 2 Department of Obstetrics, Gynaecology and Oncology, Collegium Medicum Nicolaus Copernicus University, Ujejskiego 75, 85-168 Bydgoszcz, Poland; 3 Department of Tumor Pathology and Pathomorphology, Oncology Centre - Prof. Franciszek Łukaszczyk Memorial Hospital, 85-796 Bydgoszcz, Poland; 4 Chair of Pathology, Dr Jan Biziel Memorial University Hospital, 85-168 Bydgoszcz, Poland; 5 Institute of Human Genetics, Polish Academy of Sciences, 60-479 Poznan, Poland; 6 Chair of Oncologic Pathology and Prophylaxis, Poznan University of Medical Sciences, Greater Poland Cancer Center Poznan, Poznan, Poland

**Keywords:** Alginate encapsulation, Cell block, Cytology, Cytopathology, Cancer, Cancer, Cancer models, Cancer, Diagnostic markers

## Abstract

Cell blocks help preserve cytological material and facilitate ancillary diagnostics, but their accuracy is often compromised by uneven cell distribution and interference with immunohistochemical staining. We present an internally validated protocol for alginate-encapsulated cell blocks, which have been successfully used in routine pathology and during cancer research studies. Harvested cells were suspended in phosphate-buffered saline and centrifuged. Cell pellets were mixed with an alginate solution, immersed in 0.1 M calcium chloride to form conglomerates, fixed in 10% buffered formalin, processed in a tissue processor, embedded in paraffin, sectioned, and subjected to routine diagnostic protocols. Implementing alginate-encapsulated cell blocks in our laboratory allowed for the evaluation of pleural effusion, peritoneal effusion, and bronchial washings, with the cytopathological diagnoses matching the histopathological diagnoses in over 95% of cases analyzed. Cell blocks were internally validated against results obtained from histopathologic specimens and did not require manufacturer-provided kits. We have successfully applied the protocol to characterize the newly established mucoepidermoid carcinoma cell line (IHG-MUC360) expressing GOPC, with potent use in in vitro and in vivo studies. Integrating alginate encapsulation and cell block preparation significantly advances cytological diagnostics. Alginate-encapsulated cell blocks provide a cost-effective alternative to traditional methods, allowing long-term storage and re-evaluation of prepared samples. These findings are based on single-center internal experience and warrant further validation in larger, multicenter cohort.

**Supplementary Information:**

The online version contains supplementary material available at 10.1038/s41598-025-21615-0.

## Introduction

Due to its availability and rapid processing, cytological material constitutes a cornerstone of pre-operative pathological diagnosis^1,2^. However, many traditional cytological preparation methods have limitations for further processing and ancillary studies^3,4^. Adapting alternative preparation methods to pathological practice has partially addressed those shortcomings^1^. Among them, cell blocks, a paraffin-embedded version of cytology specimens, have recently gained attention due to their ability to preserve cellular architecture and enhance the reliability of diagnosis^2^. Compared with formalin-fixed paraffin-embedded tissue specimens (FFPE), cytological specimens are fixed mainly in alcohol and, thus, are considered more advantageous in preserving the proteins and the nucleic acid. Cytological specimens provide a valuable resource for pathological examination, including immunocytochemical panels and molecular evaluation. However, pre-analytical precautions, such as appropriate protocols, fixatives, and preparatory techniques, are crucial.

Alginate-based hydrogels are the most commonly used medium for cell encapsulation, valued for their biocompatibility, low toxicity, and ability to form three-dimensional structures^5^. They are typically produced by combining sodium alginate with deionized water and inducing ionic cross-linking through the addition of calcium ions. This process results in a stable hydrogel matrix capable of supporting isolated cells in both in vitro and in vivo applications^6,7^. Owing to these properties, alginate-based systems are easy to handle and can be processed using automated equipment^8^.

Despite these advantages, the application of alginate-based encapsulation in diagnostic pathology has been limited. Classic protocols often interfere with histochemical staining and lack structural stability, which restrict long-term storage and routine implementation^8,9^. In standard procedures, cells are suspended in alginate solution, polymerized with calcium ions, and subsequently embedded in paraffin to allow histological sectioning and auxiliary diagnostics.

To overcome these limitations, we developed and validated a protocol for alginate-based cell blocks, which has been successfully applied in our pathology department for both routine diagnostics and cancer research. Its implementation in clinical practice supports comprehensive assessment of cytological material, enhances diagnostic reliability, and offers a cost-effective solution suitable for research settings. This study aimed to present the alginate encapsulation technique for cell block preparation, with particular attention to morphological preservation, reduction of processing artifacts, and compatibility with immunohistochemical (IHC) staining.

## Materials and methods

Macroscopic evaluation of the material.

1. Note the date and time (hh: mm) of material delivery.
2. Check the date and time of material collection.
3. Check the date and time of material fixation.
4. Note the material quality, volume, density, transparency, and color.

### Processing of cytological specimens

#### Processing initiation—handling of the cytological specimens after collection

Processing of cytological specimens collected by fluids, liquid-based cytology (LBC), fine-needle aspiration, etc.:

1. Cytological specimens should be processed as soon as possible to prevent cellular degradation and ensure optimal diagnostic yield.
2. If it is not possible to process the material immediately after collection, store the cytological specimens suspended in 10% neutral buffered formalin (NBF) in a refrigerator (4 °C), and they must be delivered to the pathology department as soon as possible.
3. Avoid leaving unprocessed cytological material at room temperature (approximately 25 °C) in places with direct sunlight, high temperatures, and high humidity.
4. LBC specimens must be treated with an LBC preservative solution as soon as possible after collection.
5. LBC samples must be stored at room temperature or in a refrigerator (4 °C), avoiding places with direct sunlight, high temperatures, and high humidity.

#### The pre-analytical stage/or analytical stage in case of cytological material not fixed after collection

1. If the material volume exceeds 100 ml, gently shake the material.
2. Collect 30 ml of fluid, ensuring the preservation of any tissue fragments.
3. Mix the fluid with 10% buffered formalin at pH 7.2–7.4 in a 1:1 ratio.
4. Process the specimen immediately. If this is not possible, place the fixed material in a refrigerator (4–8 °C) and process as soon as possible.
5. Total fixation time for cytological material: 6–24 h, storing conditions 4–8 °C.

### Preparation of blocks of alginate-encapsulated cells

1. After fixation time, remove the material from the refrigerator and leave it for 20 min to reach room temperature without shaking or mixing it.
2. Evaluate the cell pellet appearance.

#### The cell pellet is visible, and the cell pellet volume is ≥ 0.5 ml

**Fig. 1:**
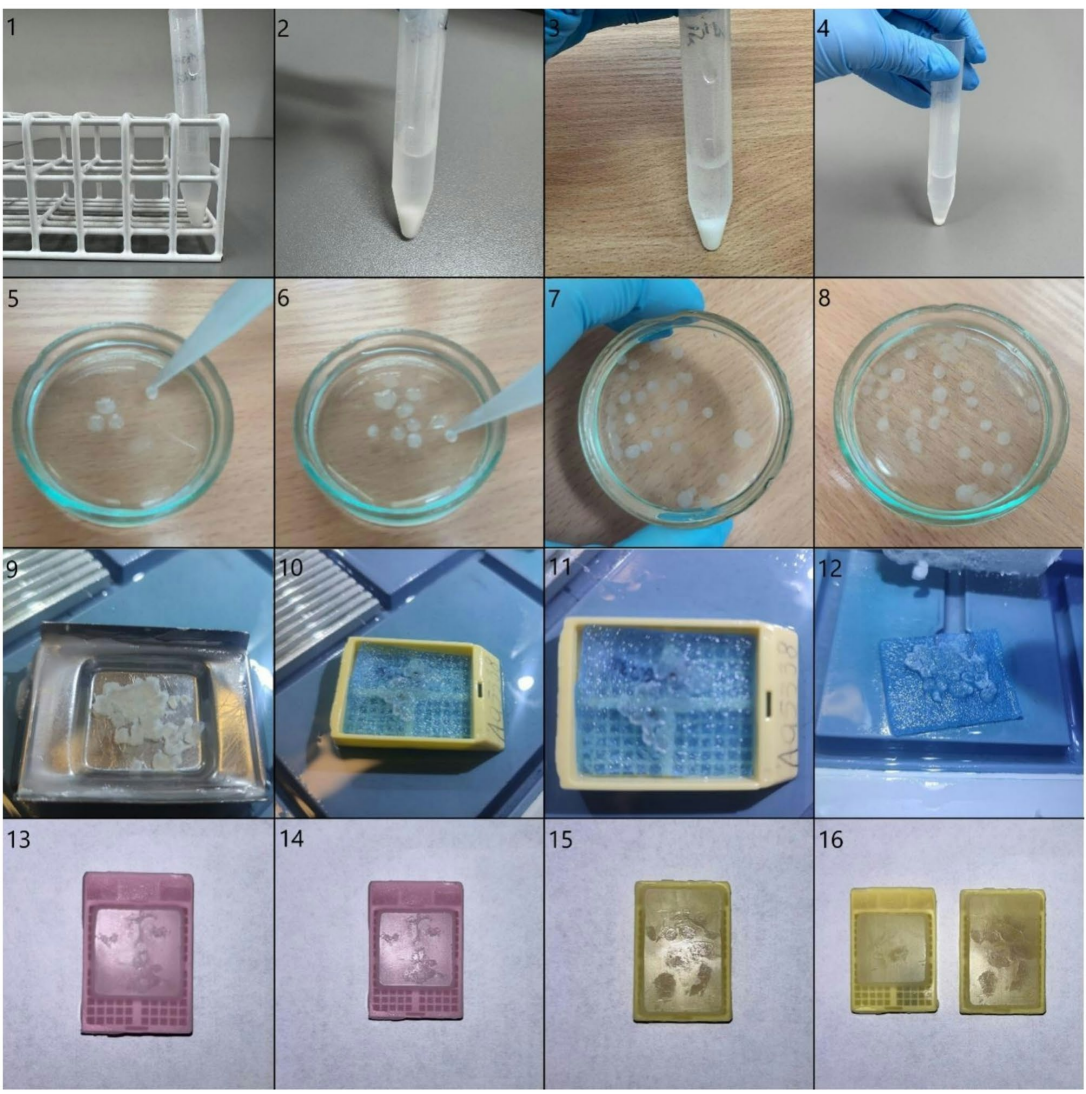
Sequence of cell block preparation creation. Cytological material in the form of cell pellets was obtained using the BD Sure Path method and suspended in a preservative fluid (1–4). Then, 1% alginate solution was added, and the mixture was slowly dripped into 0.1 M calcium chloride solution to form spherical suspensions (5–8). The received alginate encapsulates were moved to a special casket, and the automated slides preparation protocol was initiated (9–12). Finally, the encapsulates were fixed with formalin, cooled down, and cell blocks were formed (13–16).

- If the cell pellet is clearly visible and the cell pellet volume is ≥ 0.5 ml:
  pour off the supernatant into a new centrifuge tube (do not utilize the supernatant);
  add approximately 750 µl of 1% alginate sodium to the cell pellet.
- Concentrate the supernatant by centrifugation:
  centrifuge the material for 10 min at 2500 rpm;
  pour off the supernatant and add approximately 200 µl of 1% alginate sodium to the cell pellet.
- Gently mix with a pipette tip.
- Pipette the suspension and slowly drip it into 0.1 M calcium chloride solution to form spherical suspensions.
- Incubate in the 0.1 M calcium chloride solution for 15 min.
- Pipette the alginate beads into a histopathological cassette, securing the material on a biopsy sponge at the bottom and top (Fig. 1).
- Perform routine diagnostic processing in a tissue processor, form paraffin blocks, and section on a microtome.

#### The cell pellet is visible, and the cell pellet volume is < 0.5 ml

- If the cell pellet is not visible and the cell pellet volume is < 0.5 ml:
  vortex briefly to mix the solution;
  pour 10 ml into a new centrifuge tube;
  centrifuge the material for 10 min at 2500 rpm, and decant the supernatant.
- Pour off the supernatant and add approximately 750 µl of 1% alginate sodium to the cell pellet.
- Gently mix with a pipette tip.
- Pipette the suspension and slowly drip it into 0.1 M calcium chloride solution to form spherical suspensions.
- Incubate in the 0.1 M calcium chloride solution for 15 min.
- Collect the alginate beads with a pipette into a histopathological cassette, securing the material on a biopsy sponge at the bottom and top.
- Perform routine diagnostic processing in a tissue processor, form paraffin blocks, and section on a microtome.

### IHG-MUC360 cell line

The described protocol was used to establish a novel IHG-MUC360 cell line (patent number: 2018; PAT. PL 229 507 B1) – the first mucoepidermoid carcinoma cell line with a clonal reciprocal translocation t(5;7)(p15;q32). The cell line was established in-house by co-authors at the Institute of Human Genetics Polish Academy of Sciences (Poznań, Poland) using patient-derived mucoepidermoid carcinoma tissue. It is not commercially available.

**Fig. 2:**
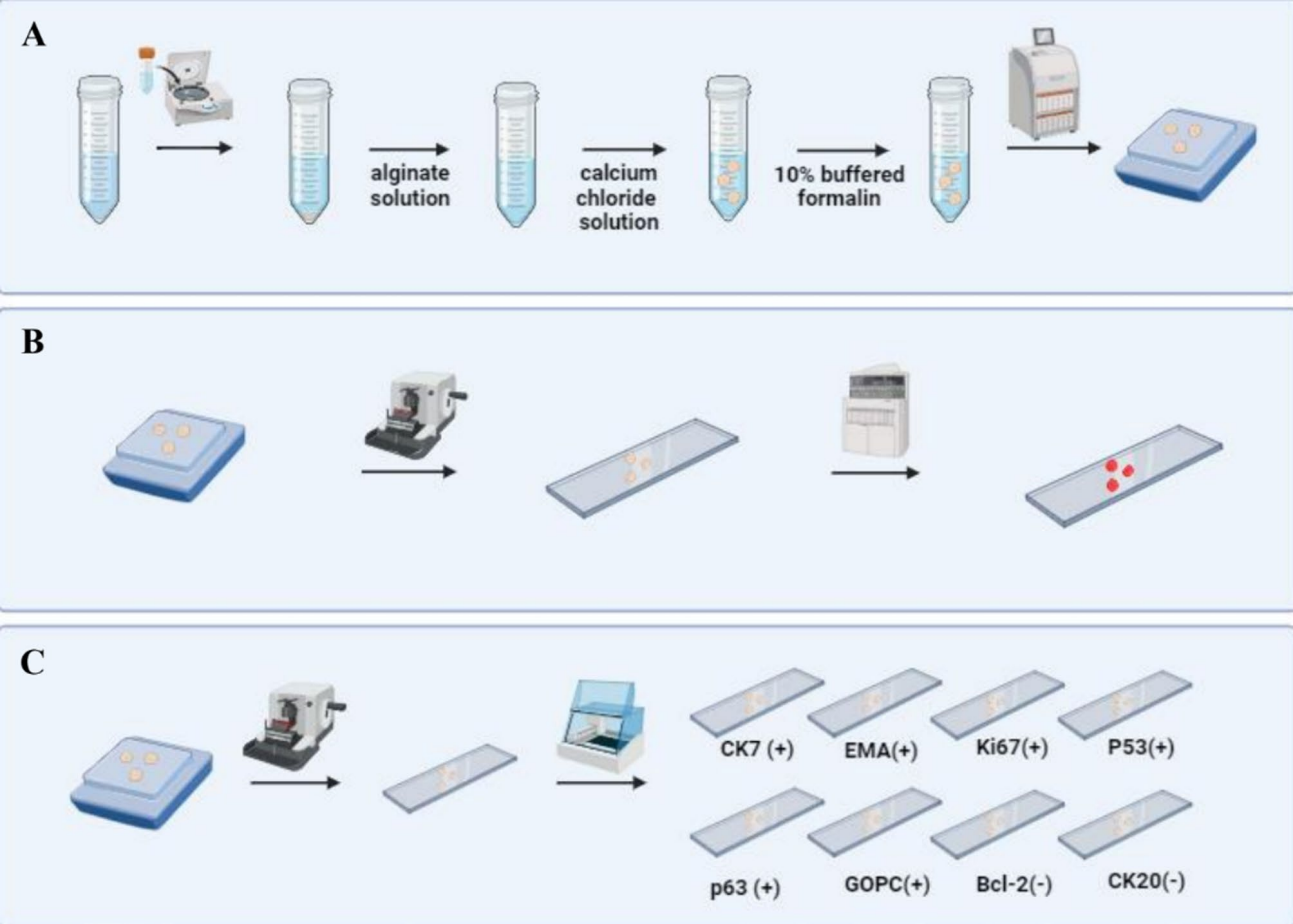
Steps for alginate-encapsulated cell block preparation for in vivo studies on the example of IHG-MUC360 mucoepidermoid carcinoma cell line. (**A**) The cell suspension underwent centrifugation, and an alginate solution and calcium chloride solution were subsequently introduced. The resulting conglomerates were fixed using a 10% buffered formalin solution and Duboscq fluid. Then, they were processed through a routine diagnostic procedure in a tissue processor and embedded to create paraffin blocks. (**B**) The cellular material embedded in paraffin was sectioned into 4 μm-thick slices using a hand-held rotary microtome. These paraffin sections were applied to basal slides, and automated hematoxylin-eosin staining was performed. (**C**) The test material was sliced into 3 μm-thick sections using a hand-held rotary microtome and placed on Superfrost Plus basal slides with increased adhesion. An automated immunohistochemical staining procedure was conducted. The cell line showed positive expression for CK, EMA, Ki67, P53, p63, and GOPC while also not expressing Bcl-2 and CK20.

First, the collected cells were suspended in Phosphate Buffered Saline (PBS), collected by centrifugation, mixed with alginate solution (catalog number: 2023; 50K0180), and immersed in a calcium chloride solution (catalog number: 2023; 21097-250G) to form conglomerates, fixed using a 10% buffered formalin solution. After fixation, the conglomerates were processed in a tissue processor and embedded in paraffin [Figure 2a]. For 3D culture, alginate beads were formed by adding 1% alginate solution to adipose-derived stem cells. The cell blocks were then sectioned into 4 μm-thick slices on a hand-held rotary microtome, applied to basal slides, and stained with H&E [Figure 2b]. Finally, the material was sliced into 3 μm-thick sections, placed on Superfrost Plus basal slides with increased adhesion, and underwent automated immunohistochemical staining [Figure 2c]. In this experimental setting, fixation with 10% buffered formalin was performed after the formation of alginate conglomerates. This sequence differs from the workflow used for clinical cytological specimens (Sect. 2.1.1), where fixation typically precedes alginate encapsulation due to the need for immediate preservation and unknown cellularity. In controlled laboratory conditions with predefined cell content, post-encapsulation fixation allows for optimal bead formation and structural preservation.

### Histochemical assays

The paraffin-embedded cellular material was cut into 3 μm-thick slices on an automatic rotary microtome. The paraffin sections were then applied to basal slides, and automated H&E staining was performed (Supplementary Tables 1 and 2). H&E staining of paraffin-embedded slice preparations showed representative mucoepidermoid cancer in each of the analyzed cell pellets obtained from passages 0, 1, and 4. Depending on the sample size, long or short processing protocols were initiated. We found no significant differences in the quality of the preparations using both methods.

### Immunohistochemical assays

The material was sliced on an automatic rotary microtome into 3 μm-thick sections and placed on a Superfrost Plus adhesion-enhanced basal slide. Immunohistochemical staining was performed automatically using Dako’s AutostainerLink48 and Benchmark ULTRA (Roche, Ventana) following the manufacturer’s protocol. Before being examined, the samples were deparaffinized and progressively dehydrated using a series of progressive ethanol concentrations (80%, 90%, 96%, and 99.8%), cleaned in xylene (steps I to IV), and mounted with a mounting medium. Then, cell pellets obtained from passages 0, 1, and 4 were examined. The panel of antibodies used to determine the characteristics of mucoepidermoid carcinoma cells is described in Supplementary Table 3.

## Results

### Alginate encapsulation cell blocks in routine cytopathological practice

The procedure described for alginate encapsulation was incorporated into our department in 2016 and has been successfully utilized in routine diagnostics. In our single-center experience, more than 95% of cytological diagnoses obtained with alginate-encapsulated cell blocks were consistent with corresponding histopathological and clinical diagnoses. Although detailed statistical analyses (e.g., kappa statistics or confidence intervals) and specimen-type stratification were not performed, the observed concordance indicates potential diagnostic utility. These findings should be interpreted as preliminary and warrant confirmation in larger, multi-institutional cohorts. The following section will present exemplary cases of alginate-encapsulated cell blocks prepared from various types of cytological material and used in routine clinical practice.

#### Bronchoalveolar lavage

**Fig. 3:**
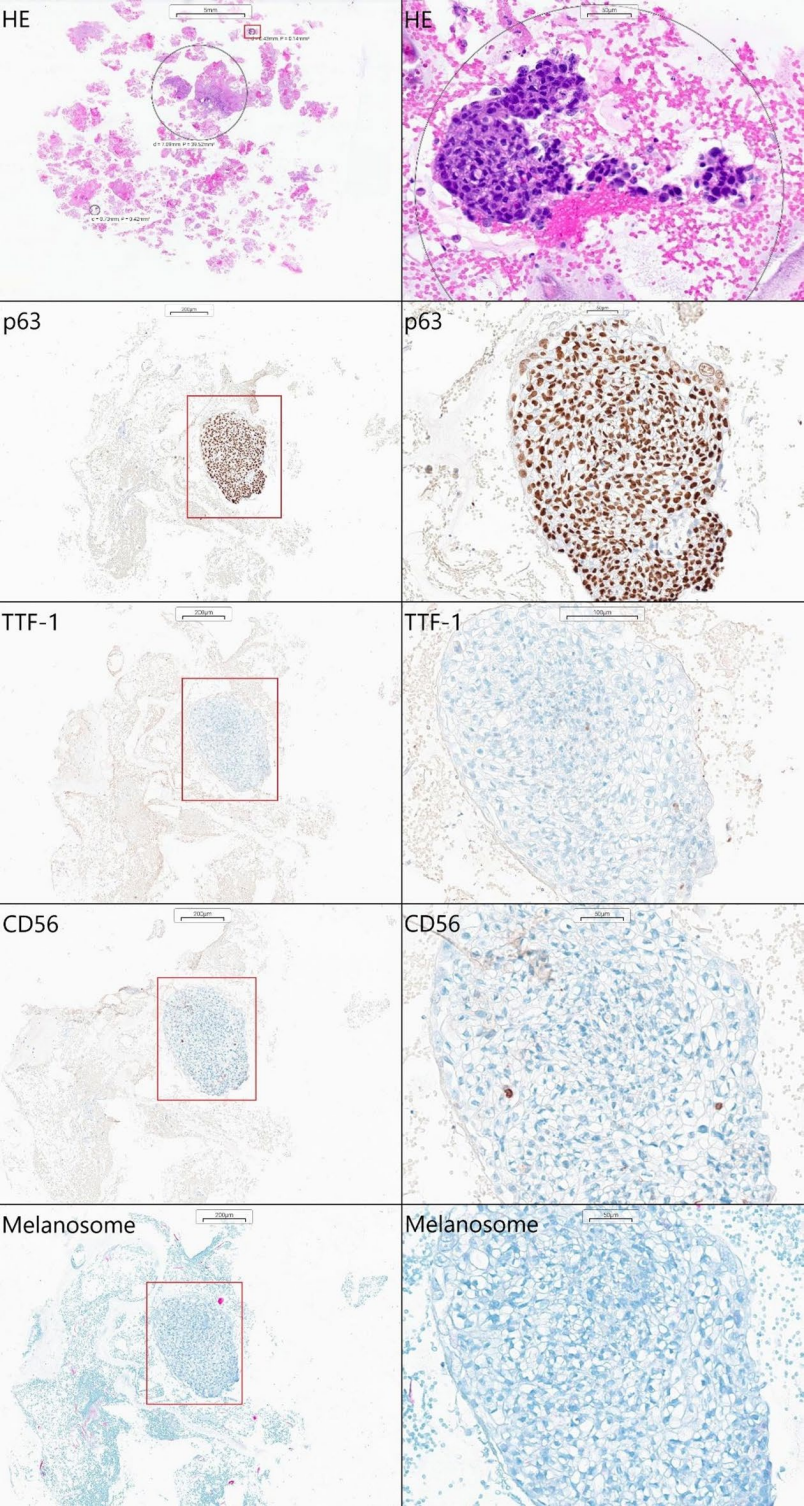
Lung cancer diagnosed using encapsulation-based cell blocks. The coexistence of p63 expression with negative TTF-1, CD56, and melanosome staining indicates a squamous cell subtype. Positive cells are stained brown.

Bronchial washings collected during bronchoscopy from an 85-year-old woman with a suspicion of lung cancer were sent to our lab for routine diagnostics. The initial cytological examination revealed the presence of poorly differentiated lung cancer cells [Figure 3]. Subsequent alginate-encapsulated cells, prepared following the described protocol (Sect. 2.1), were sectioned and stained for p63, TTF-1, CD56, and melanosomes following the current guidelines^10–12^. Only p63 demonstrated notable expression, indicating a squamous cell lung cancer diagnosis.

#### Pleural effusion fluid

**Fig. 4:**
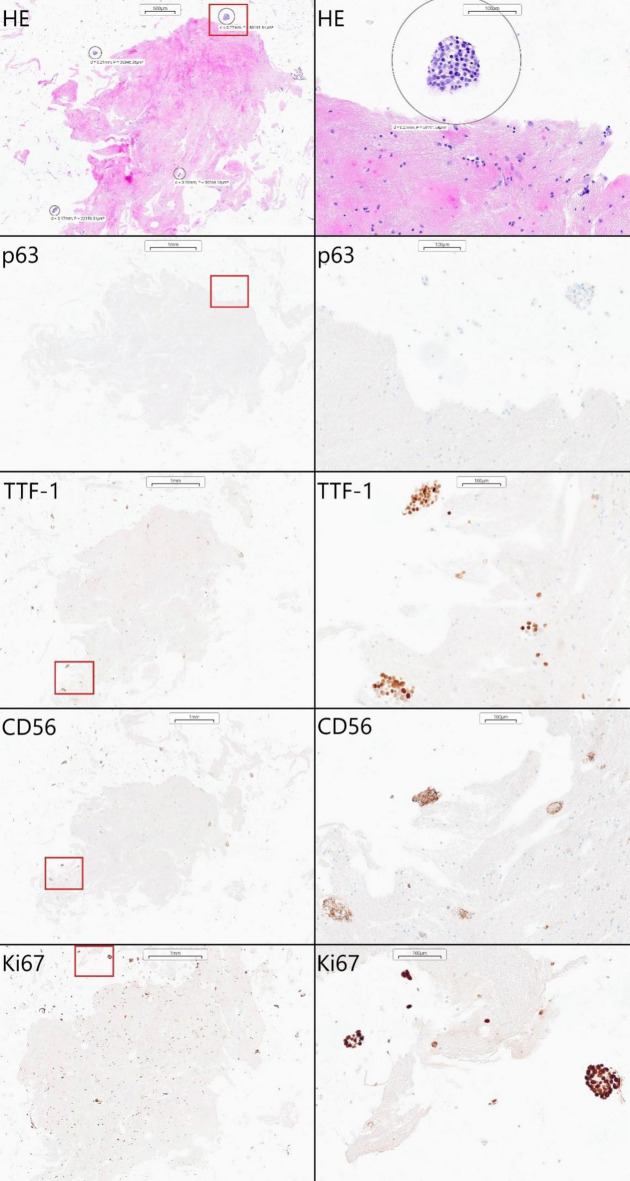
Small-cell lung cancer diagnosed using encapsulation-based cell blocks. Examination revealed characteristics of small, round, blue cells, which were negative for p63, focally positive for TTF-1 and CD56 staining, and had a high mitotic index (Ki67 over 90%). Positive cells are stained brown.

Pleural effusion fluid collected during the thoracocentesis from the right pleura of a 74-year-old woman was delivered for routine cytological examination. Initial analysis revealed the presence of small, round, blue cells characteristic of neuroendocrine malignancies. The rest of the material was prepared as an alginate encapsulation cell block, and the subsequent immunochemistry showed a focal expression of CD56 and TTF-1 with negative p63 staining [Figure 4]. Given the high mitotic index (Ki67 over 90%), the findings were sufficient to diagnose small-cell lung cancer (SCLC).

#### Peritoneal effusion fluids

**Fig. 5:**
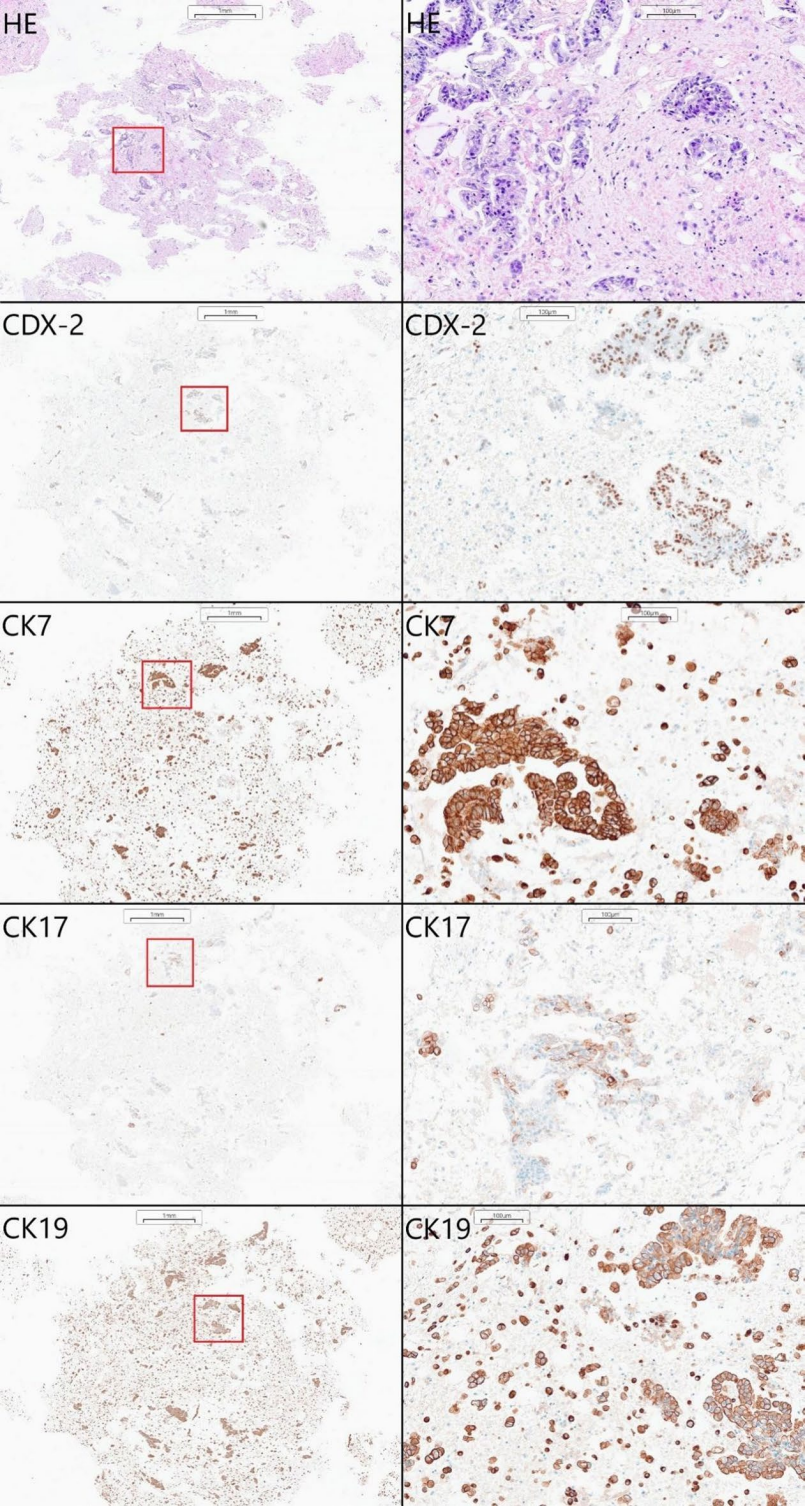
Metastatic adenocarcinoma with the probable primary site in the gastrointestinal tract diagnosed using encapsulation-based cell blocks. The examined cells showed positive CK7, CK17, CK19, and CDX-2 staining. The positive cells are stained brown.

While the described methodology was used in our laboratory primarily to diagnose lung malignancies, it also showed satisfactory results when other cancers were involved. In one such case, we received peritoneal effusion fluid collected by paracentesis from a 61-year-old woman with ascites of unspecified origin. The initial cytological examination revealed adenocarcinoma cells, which were not otherwise specified [Figure 5]. The remaining material was processed into an alginate-encapsulated cell block, sliced, and stained, revealing cells that were positive for CDX-2, CK7, CK17, and CK19. This immunophenotype was the most specific for a malignancy primarily localized within the gastrointestinal tract, including the hepato-pancreatic area. Clinically, a metastatic cancer with a secondary peritoneal involvement was diagnosed.

### Alginate-encapsulated cell blocks in in vitro studies

**Fig. 6:**
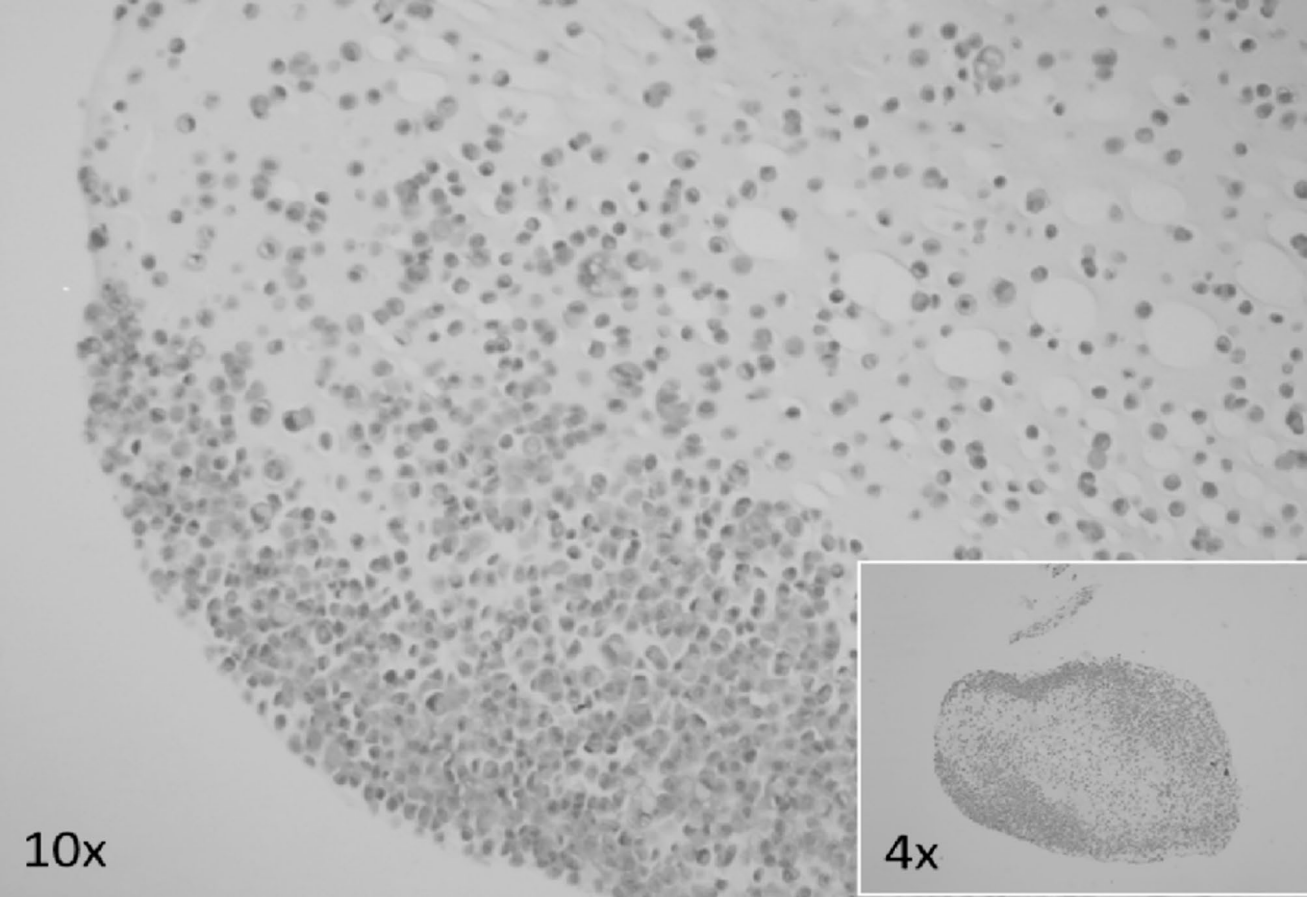
Alginate-encapsulated cell block slide containing IHG-MUC360 mucoepidermoid carcinoma cells stained with hematoxylin-eosin.

The described methodology was utilized to characterize the newly established IHG-MUC360 cell line (patent number: 2018; PAT. PL 229 507 B1) and GOPC protein expression [Figure 6].

**Fig. 7:**
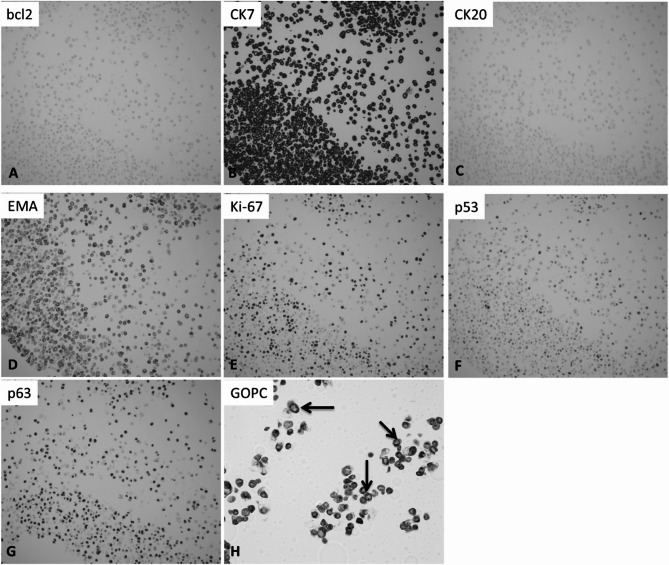
Representative immunohistochemical staining of alginate-encapsulated cell block slides containing the IHG-MUC360 mucoepidermoid carcinoma cell line. The analyzed material lacked the expression of Bcl-2 and cytokeratin 20 (CK20). The expression of CK7 was observed in all examined cells. In contrast, EMA expression was present in ≥ 90% of cells, Ki-67 expression in approximately 50% of cells, p53 expression in about 20% of cells, p63 expression in about 50% of cells, and membranous-cytoplasmic expression of GOPC in all cancer cells. Positive reactions are indicated by brown staining, with cell nuclei counterstained with hematoxylin. Primary lens magnification was 20x for GOPC and 10x for other stains.

The developed mucoepidermoid cell line showed positive expression for CK, EMA, Ki67, P53, p63, and GOPC while lacking Bcl-2 and CK20 expression [Figure 7]. The analyses confirmed that the tumor cells are functional and show a staining pattern characteristic of mucoepidermoid carcinomas.

## Discussion

Cell blocks remain a cornerstone of routine pathological diagnostics, allowing for the preservation of tissue microstructure and enabling precise diagnosis prior to surgery^13,14^. While direct smears and liquid-based biopsies offer good cytomorphological detail, only cell blocks preserve small tissue fragments and provide insight into overall architecture^15,16^. Once embedded in paraffin, they can be sliced and subjected to ancillary diagnostics, including immunohistochemical and molecular studies^3,4,17^.

The preparation process typically involves several steps, including material collection, centrifugation, paraffin embedding, and tissue staining^3,13,14^. Two main ways of preparing cell blocks include adding plasma thrombin reagent to a centrifuged cytology specimen or using a 2% agar solution to form an agar block^18,19^. Beyond these, various other methods have been described — including gel-based (gelatin, agar, HistoGel™), enzymatic (plasma/fibrinogen-thrombin), and chemical coagulation (egg albumin-alcohol, alginate-calcium). The Shidham method (NextGen CelBloking™) is also used in some settings; however, due to differing limitations, no single gold standard has been established^13,20,21^.

Moreover, traditional methods often suffer from uneven cell distribution, inadequate fixation that distorts morphology, or the use of components that interfere with immunostaining^1,9^. These issues can compromise diagnostic reliability and reduce the utility of cell blocks in clinical practice. Even when the diagnostic quality is sufficient, practical aspects of cell block preparation — such as time, cost, and processing workflow — can still be a barrier to wider adoption. Preparing a cell block is both time-consuming and costly, which can delay diagnostic turnaround. To address this, many laboratories process part of the cytological specimen as a conventional smear, while reserving the remaining material for cell block embedding^19,22^. Although this dual approach may initially increase the workload, it helps preserve material and allows for additional testing if needed. Automated systems such as Cellient™ can reduce manual labor and accelerate processing, but their upfront cost, reliance on proprietary fixatives, and limited throughput (one block at a time) often restrict their use in routine settings^13,15,23,24^. These limitations underscore the need for alternative cell block techniques that are low-cost, technically accessible, and adaptable to different laboratory settings.

Recently, Kirbis and colleagues surveyed over 400 European laboratories regarding challenges in the preparation and use of cell blocks in routine cytopathological practice. The most common problems reported included low cellularity (62%), uneven cell dispersion (22%), and insufficient sectioning of cell blocks (15%). Among the different techniques, albumin-based preparations stood out as the least reliable, often yielding poor-quality blocks and inconsistent diagnostic results. By contrast, gelatin- and agar-based methods appeared to perform more consistently^19^. A concise summary of the reported issues is provided in Table 1.

**Table 1:** Common pitfalls in traditional cell block preparation in 402 European laboratories (based on Kirbis et al., 2024).

Issue	Prevalence	Most affected methods	Impact on diagnosis	Probable causes	Mitigation strategies
Low cellularity	62% of labs (248/402)	Collodion (100%)^†^, Gelatin (75%), HistoGel (69%)	Risk of false negatives; material loss	Suboptimal pellet formation, poor triage	Improved sample triage (cell count); 3D encapsulation
Dispersed cells	22% of labs (89/402)	CytoFoam (100%), albumin (50%), gelatin (50%)	Difficult H&E interpretation	Matrix structure fails to retain cell cohesion	Use of gels with better cohesion; alginate matrix
Insufficient sectioning	15% of labs (61/402)	Albumin (25%) Cell scrape (20%), Cellient (15%)	Limits re-evaluation and IHC	Poor block orientation; small cell pellets	Embedding in sponge; serial sectioning
Inconsistent results	10% of labs (41/402)	Albumin (25%), Cell scrape (20%), Cellient (15%)	Unreliable ancillary testing	DNA contamination (e.g. plasma), fixative issues	Avoiding thrombin; validated fixative protocols
Poor morphology or antigenicity loss	8% of labs (6/402)	Albumin (50%), Cell scrape (28%), Cellient (15%),	Affects subtype classification, IHC	High processing temperature, overfixation	Use of low-temp gels (e.g. HistoGel), formalin optimization

^†^Small number of laboratories using this method may lead to interpretation bias.

The data summarized in this table are based on the findings reported by Kirbis et al., Cancer Cytopathology 2024;132(4):250–259. The structure, interpretation and proposed mitigation strategies reflect the authors’ own analysis.

Many of the issues in the survey also affected our laboratory in the past. However, the implementation of alginate-based encapsulation helped resolve several of them^25,26^. Unlike conventional methods, where cells often accumulate at the bottom of the block, alginate encapsulation enables a uniform distribution throughout the paraffin Sects^7,25^. This reduces the risk of losing diagnostic material during slicing and improves the evaluation of samples with low cellularity^27^. Moreover, the encapsulated material can be safely archived and reused if needed. The method integrates well into routine workflows and offers a cost-effective alternative to traditional preparation techniques^13,22^. To contextualize these advantages, we compared alginate encapsulation with other commonly used cell block methods in terms of section quality, technical requirements, and diagnostic compatibility. A detailed comparison is provided in Table 2.

While alginate encapsulation integrates well with routine paraffin workflows and does not require proprietary kits, the method currently relies on manual handling and timely embedding to prevent drying artifacts. These characteristics may limit its immediate scalability in high-volume settings. However, the consistency of section quality and procedural simplicity suggest that the method could be further optimized for semi-automated or standardized formats. Our group’s ongoing work focuses on refining pellet formation and evaluating batch processing potential.

**Table 2:** Comparative overview of cell block preparation techniques.

Method	Cellularity	Section consistency	Artifacts	Cost & workflow	Key features & limitations
Plasma–thrombin	Moderate-high	Variable (clot-dependent)	Low	Low cost; simple manual process	Readily available; uneven cell distribution; plasma may introduce DNA contamination; not formalin-compatible
Agar / HistoGel™	Moderate-high	Uneven (sedimentation)	Low	Moderate; easy to handle; relatively quick; high workloads	Sedimentation at block base; gel shrinkage; limited archival stability
Alginate (manual)	Moderate	Moderate (bottom-loading)	Low–moderate	Manual; paraffin-compatible	Easy to implement; may require optimization; uneven cell distribution without encapsulation
Alginate encapsulation	High	Excellent	Moderate (if delayed embedding)	Manual; paraffin-compatible; moderate cost	Uniform 3D cell distribution; consistent sections; compatible with IHC/molecular tests; requires prompt embedding
Collodion bag	Moderate	Good	Moderate	Manual; time-consuming	Excellent for hypocellular samples; suitable for IHC and molecular studies; technique-sensitive; not widely adopted;
Cellient™	Variable	Excellent	Low	High cost; automated; shorter preparation time	Requires proprietary reagents; limited throughput; expensive setup
NextGen™ (Shidham)	High	Excellent	Low	Manual, standardized kits	Requires commercial kit; reduced procedural flexibility

*IHC* immunohistochemistry.

In addition to the preparation technique itself, several preanalytical factors can influence cell block quality, including the nature of the lesion, type of fixative, and handling protocol. For example, while formalin fixation is essential for immunochemistry, it can degrade DNA and alter antigenicity, limiting its suitability for molecular assays^1,20^. In specimens like serous fluid or fine-needle aspirates, where cells are often scattered or scant, cytotechnologists may find it difficult to locate diagnostically relevant areas^25,28^. In such cases, the use of alignment and verification (AV) markers can improve sectioning accuracy and minimize material loss, but this also increases the cost and time to diagnosis^13^.

In our department, we adopted a specific protocol to standardize the handling of cytological material in cases of suspected malignancy. Upon sample collection, a portion of the material is used for a direct smear, while the rest is encapsulated in alginate, fixed with formalin, and embedded in paraffin to form long-lasting cell blocks. These blocks are routinely sliced, stained with H&E, and subjected to cytopathological examination. In most cases, particularly lung cancers, the diagnosis is made based on characteristic cytopathological features. If ancillary studies are needed, further slices are stained with a limited immunocytochemical panel (TTF-1, p63, CD56, Ki-67) to distinguish between SCLC and NSCLC subtypes. The material can also be preserved for future molecular testing. Importantly, due to the even distribution of cells throughout the block, all sections are diagnostically equivalent.

This approach is especially beneficial when biological material is scarce or when invasive procedures (such as transbronchial biopsy) are contraindicated. For example, bronchial washings often yield limited cellularity, which may suffice for detecting neuroendocrine features but not for differentiating histological subtypes of SCLC and NSCLC^10^. In such cases, cell blocks improve diagnostic yield and enable ancillary testing. They have demonstrated superior sensitivity and specificity compared to cytological smears, particularly in fine-needle aspirates and pleural or peritoneal effusions^29–31^. Moreover, cell blocks allow for retrospective re-evaluation or molecular profiling in the event of disease progression, potentially obviating the need for repeated sampling^10^.

Beyond routine diagnostics, alginate-based encapsulation has also proven useful in experimental and translational research. In our laboratory, this method was used to visualize the ELF1 transcription factor in lymphoma cell lines by IHC, demonstrating its applicability in cancer research^32^. Since the procedure does not rely on proprietary kits and can be validated internally, it offers transparency and reproducibility, features increasingly expected in modern research settings. Furthermore, the ability to generate multiple sections from a single block facilitates repeated analyses, minimizes material waste, and allows researchers to monitor consistency across different staining protocols.

Taken together, alginate encapsulation offers a practical and scalable platform for research applications, particularly when material availability is limited or when long-term storage and re-evaluation of archived samples are required.

To further assess the molecular compatibility of the encapsulated material, we are currently analyzing the integrity of DNA and RNA extracted from alginate-encapsulated cell blocks for potential application in next-generation sequencing (NGS). This ongoing effort aims to determine whether the method preserves nucleic acids at a quality sufficient for advanced molecular diagnostics.

## Conclusions

Alginate-based cell encapsulation offers a reproducible and technically simple approach to preparing cell blocks from cytological specimens. In our experience, this method improved section quality, reduced material loss, and enabled consistent diagnostic performance across various sample types, particularly in cases with low cellularity or limited material availability. The method is compatible with both immunocytochemical and molecular assays, supports long-term material storage, and can be used in parallel with conventional smears without disrupting paraffin-based workflows. It requires no proprietary reagents or equipment and remains cost-effective even when applied routinely. Although still performed manually, its simplicity and consistency suggest that further standardization or automation could be feasible. Given that our results are based on single-center internal validation, they should be regarded as preliminary and confirmed in larger, multi-institutional studies. In our view, alginate encapsulation offers a practical alternative for both clinical diagnostics and translational applications.

## Supplementary Information

Below is the link to the electronic supplementary material.

Supplementary Material 1

## Data Availability

The data presented in this study are available on request from the Corresponding Author.
