# Supplementary material for: Alginate encapsulated cell blocks improve diagnostic consistency and long term specimen integrity in routine pathological diagnostics and cancer research
Source: Sci Rep. 2025 Oct 28;15:37603. doi: 10.1038/s41598-025-21615-0 (PMC12568939; doi:10.1038/s41598-025-21615-0)
Supplement: Supplementary file 1 — Supplementary Material 1 [file 41598_2025_21615_MOESM1_ESM.docx]

**Supplementary Table 1.** The protocols of automated slide preparation

| **Reagents** | **Long processing (time, h)** | **Short/rapid processing (time, h)** |
| --- | --- | --- |
| 10% neutral buffered Formalin | 0:40 | 0:02 |
| 70% Alcohol | 1:00 | 0:10 |
| 80% Alcohol | 1:00 | 0:10 |
| 90% Alcohol | 1:00 | 0:20 |
| 99,9% Alcohol | 1:30 | 0:20 |
| 99,9% Alcohol | 1:30 | 0:30 |
| 99,9% Alcohol | 0:30 | 0:30 |
| Xylene | 1:00 | 0:20 |
| Xylene | 1:00 | 0:25 |
| Xylene | 1:00 | 0:30 |
| Wax | 1:00 | 0:20 |
| Wax | 2:00 | 0:30 |
| Wax | 2:00 | 0:45 |

**Supplementary Table 2.** The protocol for automated H&E staining

| **Reagents** | **Time (min)** |
| --- | --- |
| Xylene | 2:00 |
| Xylene | 2:00 |
| Xylene | 2:00 |
| 99,9% Alcohol | 2:00 |
| 99,9% Alcohol | 2:00 |
| 96% Alcohol | 2:00 |
| Water Wash | 1:00 |
| Harris Hematoxylin | 7:00 |
| Water Wash | 4:00 |
| Differentiator | 0:02 |
| Water Wash | 5:00 |
| Eosin | 1:55 |
| Water Wash | 1:00 |
| 96% Alcohol | 0:30 |
| 99,9% Alcohol | 0:30 |
| 99,9% Alcohol | 0:30 |
| Xylene | 0:30 |
| Xylene | 0:30 |
| Xylene | 0:30 |
| Coverslip |  |

**Supplementary Table 3.** The characteristics of antibodies used for immunohistochemical studies.

| **Antibody (clone** | **Description** | **Catalog number (company)** |
| --- | --- | --- |
| Anti-Bcl-2 [124] | Primary, mouse, monoclonal | IR614 (Agilent) |
| Anti-CD56 [123C3] | Primary, mouse, monoclonal | IR628 (Agilent) |
| Anti-CK7 [OV-TL 12/30] | Primary, mouse, monoclonal | IR619 (Agilent) |
| Anti-CK17 [E3] | Primary, mouse, monoclonal | IR620 (Agilent) |
| Anti-CK19 [RCK108] | Primary, mouse, monoclonal | IR615 (Agilent) |
| Anti-CK20 [KS20.8] | Primary, mouse, monoclonal | IR777 (Agilent) |
| Anti-CDX-2 [ERP2764Y] | Primary, rabbit, monoclonal | 05463491001 (Roche) |
| Anti-EMA [E29] | Primary, mouse, monoclonal | IR629 (Agilent) |
| Anti-Ki-67 [30-9] | Primary, rabbit, monoclonal | 790-4286 (Roche) |
| Anti-Melanosome [HMB45] | Primary, mouse, monoclonal | GA052 (Agilent) |
| Anti-p53 [DO-7] | Primary, mouse, monoclonal | IR616 (Agilent) |
| Anti-p63 [DAK-p63]* | Primary, mouse, monoclonal | IR662 (Agilent) |
| Anti-p63 [4A4] | Primary, mouse, monoclonal | 05867061001 (Roche) |
| Anti-TTF-1 [8G7G3/1] | Primary, mouse, monoclonal | IR056 (Agilent) |
| **GOPC,** Immunogen:  [IEFEVVYVAPEVDSDDENVEYEDESGHRYRLYLDELEGGGNPGASCKDTSGEIKVLQGFNKKAVTDTHENGDLGTASETPLDDGASKLDDLHTLY] | | HPA024018 [Sigma-Aldrich] |

*used to confirm the characteristics of the IHG-MUC360 cell line
